# Effect of palmitoylethanolamide on inner retinal function in glaucoma: a randomized, single blind, crossover, clinical trial by pattern-electroretinogram

**DOI:** 10.1038/s41598-020-67527-z

**Published:** 2020-06-26

**Authors:** Gemma Caterina Maria Rossi, Luigia Scudeller, Chiara Lumini, Federica Bettio, Erica Picasso, Giulio Ruberto, Aba Briola, Alessandra Mirabile, Alessia Paviglianiti, Gian Maria Pasinetti, Paolo Emilio Bianchi

**Affiliations:** 1 University Eye Clinic, Fondazione IRCCS Policlinico S. Matteo, Pavia, Italy; 2 Clinical Epidemiology and Biometric Unit, Scientific Direction, Fondazione IRCCS Policlinico S. Matteo, Pavia, Italy; 3 Eye Unit, Istituto Beato Palazzolo, Bergamo, Italy

**Keywords:** Health care, Therapeutics

## Abstract

Glaucoma is a neurodegenerative disease, our study aimed to evaluate the potential effects of Palmitoylethanolamide (PEA) supplementation on RGCs function by PERG examination, and to record effects on intraocular pressure, visual field and quality of life. It was a single centre, randomized, prospective, single blind, two treatment, two period crossover study on stable glaucoma patients on topical monotherapy comparing current topical therapy alone or additioned with PEA 600 mg one tablet a day. At baseline, at 4 and at 8 months, all patients underwent to complete ophthalmic examination, pattern electroretinogram, visual field, and quality of life evaluation. 40 patients completed the study: mean age 66.6 ± 7.6 years; 21 (52.5%) male; 35 POAG (87.5%). At baseline, most patients had an early visual field defect, the IOP was well controlled. At the end of the PEA 600 mg supplementation, a significantly higher (mean 0.56 μV, 95% CI 0.30–0.73, p < 0.001) in the P50-wave amplitude was observed; in the PEA period a significantly lower IOP (− 1.6 mmHg, 95% CI − 2 to 1.2, p < 0.001) and higher quality of life scores (+ 6.7, 95% CI 4–9.9, p < 0.001) were observed. Our study is the first to show promising effects of PEA on PERG and on quality of life in glaucoma patients.

## Introduction

Glaucoma is a progressive and multifactorial neurodegeneration in which pro-apoptotic signals are developed towards the retinal ganglion cells (RGC) and their axons: neurodegeneration is the progressive loss of structure or function of neurons resulting from the imbalance between protective factors and harmful influences on RGCs.

It is widely recognized that the high outflow resistance in the trabecular meshwork outflow pathways causes an increase in intra-ocular pressure (IOP) and that the IOP levels cause deformation of the lamina cribrosa resulting in axonal damage and consequent apoptosis of the RGCs.

Therefore, currently, the only approach proven to be efficient in preserving visual function is lowering IOP in both the initial and advanced stages^1–4^. Many glaucoma patients experience damage that continues despite tonometric compensation, therefore other possible treatment areas have been studied, including ocular blood flow and neuroprotection.

Neuroprotection can be defined as a therapeutic approach aimed at preventing, blocking and, in some cases, reversing neuronal cell damage^5^. Numerous compounds have been shown to be neuroprotective in animal models of experimental glaucoma, such as memantine^6^ and brimonidine^7^: but so far, no compound has reached a level of evidence sufficient to be considered a neuroprotective agent in humans^8^.

Blood flow abnormalities in the optic nerve head may initiate the glaucomatous cascade leading to neuroaxonal damage^9,10^, although perfusion pressure may be relevant in glaucoma but very difficult to measure^11^. Secondary trans-synaptic degeneration may also involve higher visual centers and treatment strategies to prevent disease progression in glaucoma should also consider central neural degeneration beyond the retina and optic nerve head^12^.

Therefore, although lowering the IOP is the main strategy for the treatment of glaucoma, neuroprotection can be considered an additional therapeutic strategy targeting RGCs and neurons of higher visual centres^13^.

Palmitoylethanolamide (PEA) is classified as an endocannabinoid-like molecule; it is now available as tablets (Visimast ® 600 mg). It has been demonstrated to have at least three potential beneficial effects in patients with glaucoma.

In fact, the administration of PEA enhances aqueous humor outflow facility: this effect appears to be mediated at least partially by a SR144528-sensitive, non-CB1/CB2 receptor, the GPR55 receptor, and the PPARα receptor, and involves the p42/44 MAPK pathway. Some clinical studies have found a significant IOP-lowering effect with oral PEA 600 mg intake^14,15^. This property provides a mechanistic basis for the potential of PEA as a new therapeutic agent for the treatment of elevated IOP^16^.

Furthermore, it is able to exert the direct vaso-relaxation of the ophthalmic (bovine) artery in a time-dependent way through the transcription factors PPARα suggesting their function in the physiological mechanisms of vascular regulation^17,18^. The vasorelaxant role of endocannabinoids increases the supply of oxygen to the retina and could prevent ischemic lesions. PEA (600 mg) administered daily over a three months period improved systemic endothelial function in patients with ocular hypertension when examined using non-invasive brachial artery ultrasound assessment of flow-mediated vasodilation^19^.

Finally, some studies have highlighted the role of PEA in providing protection against neurotoxic damage to the central nervous system and eye. In vivo, PEA protects the rat's retina from ganglion cell death by activating cannabinoids receptors (CB1 and TRPV1) by modulating the glutamate-excitotoxicity and thus altering the course of apoptosis^20,21^.

To date, endocannabinoids constitute the most recent of the neuromodulators found in neural and non-neural tissues throughout the body^22^. Regarding the retina, there is general agreement that cannabinoids suppress dopamine release and presynaptically reduce transmitter release from cones and bipolar cells^21^.

Our overall aim was to evaluate the potential beneficial effects of PEA 600 mg supplementation on RGCs function in subjects with glaucoma. Our primary goal was to assess the effects of PEA 600 mg on the PERG exam at four months of therapy. The secondary objectives were to evaluate the effects on intra-ocular pressure, visual field and quality of life status.

## Materials and methods

**Figure 1:**
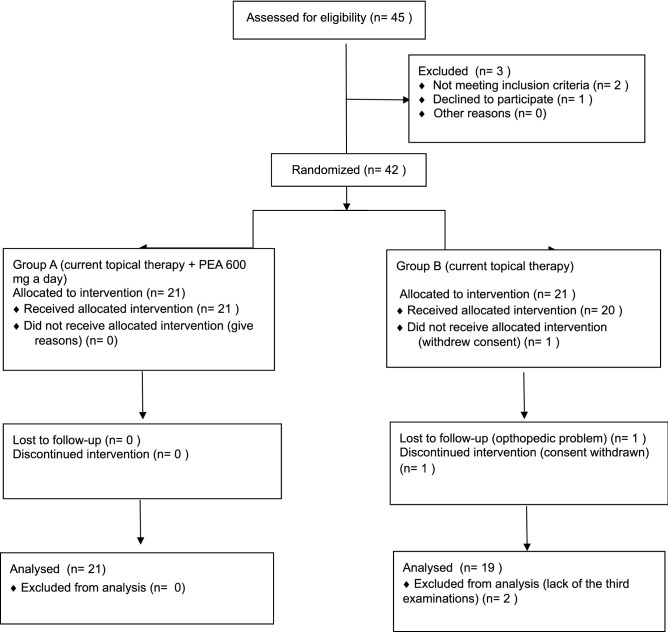
CONSORT 2010 flow diagram.

It was a single-center, randomized, prospective, single-blind, two treatment and two period crossover study conducted at the University Eye Clinic of Pavia in accordance with the Helsinki Declaration after approval by the Local Ethics Committee of the Fondazione IRCCS Policlinico San Matteo of Pavia (prot. 2015000565). The study was conducted according to the recommendations of the Helsinki Declaration (revision 2000, Edimbourg) and to the Italian Good Clinical Practice legislation (DM 15 Luglio 1997 and modifications); it complied with the CONSORT 2010 guidelines (Fig. 1) and registered on ClinicalTrials.gov (identifier NCT 04088084; 12/09/2019).

All eligible patients were invited to complete the study and to sign the informed consent form: signed informed consent was obtained from all individual participants included in the study.

The eligibility criteria were as follows: age 18 years or older; diagnosis of primary open angle glaucoma (POAG) or normal tension glaucoma (NTG) (see “Definitions”); controlled IOP (< 18 mmHg, morning value) with monotherapy with a prostaglandin analogues; stable IOP < 18 mmHg in the last 2 years; stable disease in the last 2 years (no more than -1 dB/year at MD of visual field); at least two reliable visual fields per year in the past 2 years; no filtering surgery or other ocular surgery in the previuos 6 months; written consent to participate in the study procedures and data utilization in an anonymous form.

*Exclusion criteria* ocular hypertension with normal optic nerve and visual field; contraindication to PEA; use of brimonidine eye drops; any condition limiting the patient's ability to participate in the study; other causes of visual field changes, such as cataract, myopic corioretinopathy, macular diseases, retinal vascular occlusion.

Patients who no longer wished to participate after signing informed consent or any other condition which, in the investigator's clinical judgment, made unacceptable further participation in the study for that individual patient represented withdrawal criteria.

The inclusion/exclusion criteria were verified after careful examination of the patients' medical charts. If eligible, on his/her first clinical visit after the start of the study, the patient was informed of the protocol and his/her written consent was requested.

After enrollment, at baseline, all patients underwent the following routine procedures: complete ophthalmic examination, visual acuity (VA), anterior segment evaluation (anterior chamber, lens and angle), IOP (Goldmann tonometry), pachymetry (Pachette 2 pachymeter, DGH Tecnologies, Exton, PA 19341) measurement, optic nerve evaluation with indirect lens (Volk 90), pattern electroretinogram (PERG, BM 1610, Biomedica Mangoni, Pisa, Italy), visual field examination (24–2 sita-standard, Humphrey HFA II 750 perimeter, Zeiss), assessment of the quality of life by general vision and general health scales of the Italian version of the NEI VFQ 25^23^.

Two ophthalmologists (RGCM and LC) performed VA, anterior and posterior evaluation, IOP and pachymetry. BA assisted patients in the visual field, BF in PERG. RG has analyzed PERG. RGCM, LC, BA, BF and RG were blind to patients' therapy, and randomization group.

The patients were then randomized (1:1) to group A (current topical therapy + PEA 600 mg one tablet a day) or to group B (current topical therapy) for four months. The statistician (SL) generated the random allocation sequence. One resident (PE) delivered PEA to patients based on randomization.

At Visit 1 (V1, month 4) all patients underwent all the above mentioned procedures, after which all patients were crossed-over: the patients of group A were unprescribed tablets, and group B took PEA 600 mg, one tablet a day, for 4 months.

At Visit 2 (V2, month 8) all patients underwent all the procedures described, again.

No wash-out period was performed because when the PEA is degraded, its catabolites are reused, therefore it is not subject to accumulation phenomena^24^.

We did not considered a placebo group because the patient cannot interfere with the PERG measurement which is objective and patient-independent.

### Definitions

The diagnosis of glaucoma required: IOP > 21 mmHg in at least two consecutive visits at the time of the first diagnosis for POAG, or IOP ≤ 21 mmHg in at least two consecutive visits at the time of the first diagnosis for NTG, presence of glaucomatous optic nerve head (ONH) confirmed by an expert fundus examination, and at least three reliable Humphrey 24–2 full threshold visual field tests performed on different days showing a glaucomatous or suspected glaucomatous defect.

About ocular examination, visual acuity was determined in Decimals unit; the slit lamp examination of the anterior and posterior segment was conducted with particular attention to the retina and the optic nerve aspect; the IOP measurement was assessed with Goldmann tonometry.

The Humphrey Visual Field SITA standard 24-2, a test that measures the entire area of peripheral vision that can be seen while the eye is focused on a central point, is performed quickly and easily in about 15 min. It is effective in diagnosing and monitoring the progress of glaucoma. The main parameters to evaluate the damage and the progression or not are: mean defect and pattern standard deviation (MD and PSD, decibels^25^.

**Figure 2:**
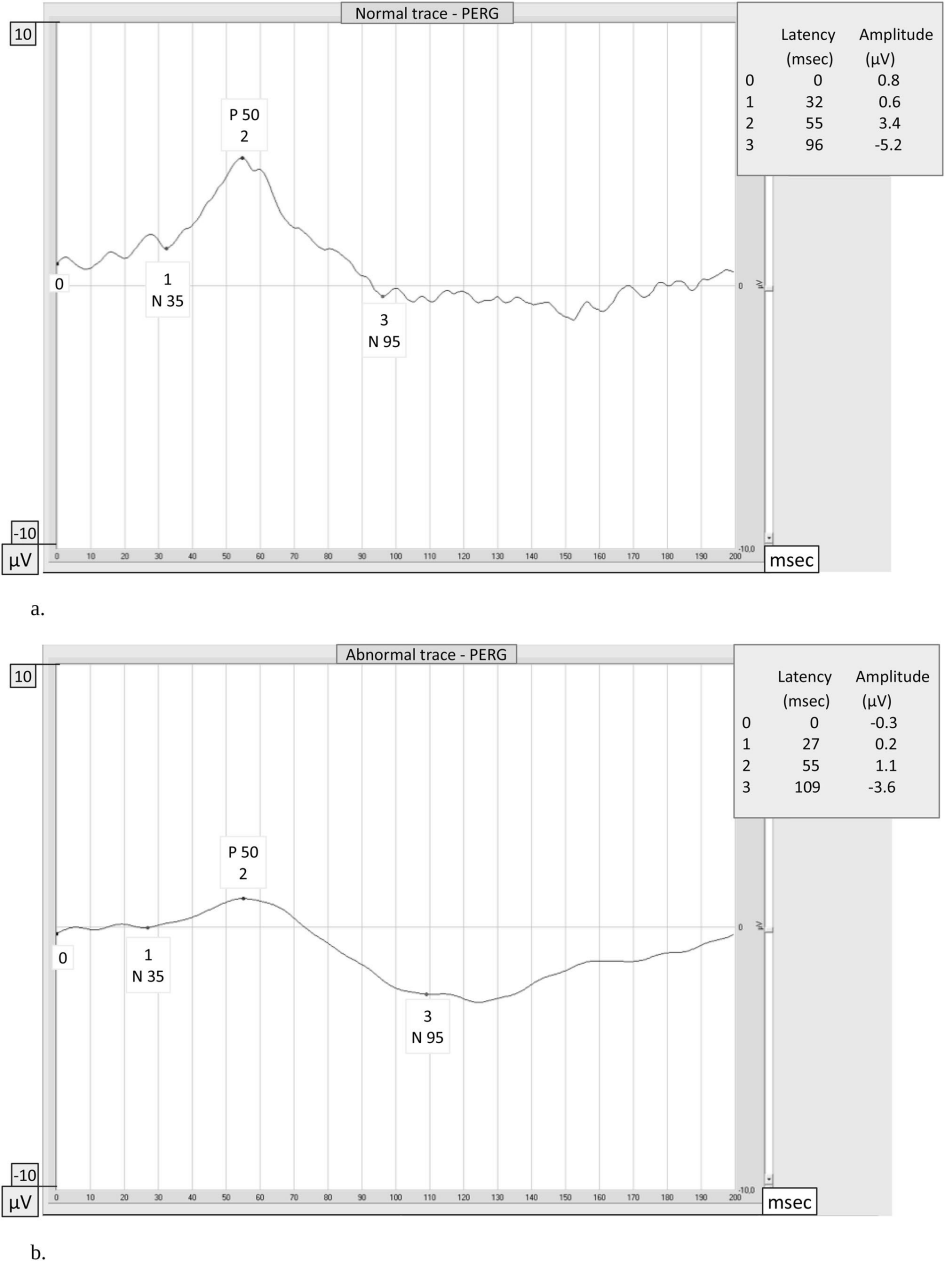
The pattern electroretinogram (PERG) waveform in normal subjects contains three components: a small negative component with a latency of approximately 35 ms (N35, #1), a much larger positive component at approximately 50 ms (P50, #2) and a large negative component at approximately 95 ms (N95, #3). Latencies are expressed as absolute values. The amplitudes are measured between peaks and troughs: the P50 amplitude from the trough of N35 (#1) to the peak of P50 (#2), and the N95 amplitude from the peak of P50 (#2) to the trough of N95 (#3). Amplitudes are expressed as differential values. (**a**) Normal PERG. In this normal subject the P50 latency is 55 ms, the P50 amplitude is 3.4 μV, the N95 latency is 96 ms and the N95 amplitude is 5.2 μV: PERG amplitudes and latencies of P50 and N95 waves are within normal limits. (**b**) Abnormal PERG. In this patient affected by glaucoma PERG amplitudes of P50 (#2) and N95 (#3) waves are reduced (1.1 μV and 3.6 μV) and latencies are delayed (55 ms and 109 ms) in comparison with normal.

The PERG is an objective and direct measure of inner retinal function related to RGCs activity^26,27^, which has been found to document and predict early glaucomatous changes. In a recent paper, Bach reported data suggesting that P50 is generated in the cell body and N95 is in the axons of RGCs in humans^28^. In addition, PERG can document, at pre-clinical stage, an improvement in the RGCs function following therapeutic IOP reduction^29^. Therefore PERG can be considered as an early indicator of functional RGC changes following an intervention aimed at counteracting apoptosis. The test records how well the cells of the retina carry electrical impulses within the eye. In particular, the P50 wave reflects the ganglion cells vitality/activity (Fig. 2a and b).

PERG was recorded according to the International Society for Clinical Electrophysiology of Vision guidelines^30^.

*Patient preparation* patient sitting 1 m away from the monitor, pupils were not dilated, appropriate refractive error correction in relation to the eye-screen distance, binocular stimulation, and monitoring with a TV camera (to stop the stimulations when frequent blinking or fixation loss) has been carried out.

*Parameters of the pattern stimulation* were as follows: 21″ CRT monitor with frame rate: 75 fps were used, a black and white reversing checkerboard was presented to the patient with a check size: 0.8°, luminance 80 cd/m^2^, contrast: 97%, temporal frequency: 4 rps (2 Hz), and central fixation was used.

*Electrodes* the DTL electrodes were used as active and a skin electrode disk was placed as reference at the ipsilateral outer canthi, with ground electrode placed on the forehead (Fpz).

*Parameters of the recording system* were as follows: filters: 1–100 Hz, switched off notch filters, analysis time: 200 ms, artefact reject threshold: 50 μV, averaging: 200 sweeps. Two consecutive waveforms were recorded, off-line averaged, and then analyzed.

Amplitude and time of the P50- and N95-waves with manual correction to the automatic cursor placement were analyzed when the automatic setting was uncertain.

The patients’ quality of life was examined with the Italian version of the 25 item National Eye Institute Visual Function Questionnaire^23^. The 25 item NEI-VFQ is a vision-targeted non-disease-specific instrument designed to measure the impact of some ocular disorders on vision related quality of life. Depending on the item, responses to this questionnaire pertain to the frequency or severity of a symptom or a problem with the functioning. The NEI-VFQ scores can range from 0 to 100 with lower scores indicating more problems or symptoms. We have considered general vision (GV), general health (GH), and the total score.

All personnel involved in the ophthalmic examination, visual field examination, optic nerve evaluation, IOP measurement and PERG test were blind to the patient’s treatment period; even those who analysed the data were blind to the treatment group.

Systemic and topical adverse events were collected.

## Study objectives

The general purpose of the study was to evaluate the potential beneficial effects of PEA 600 mg supplementation on RGCs function in subjects with glaucoma by pattern electroretinogram.

### Primary objective

To assess effects of PEA 600 mg a tablet a day on PERG examination.

### Secondary objectives

To assess effects of PEA 600 mg on IOP values, if any.

To record visual acuity, and visual field changes, if any.

To follow QL perception (GV and GH of NEI VFQ25)***.***

### Statistical issues

Sample size calculation were based on the following assumptions: calculations based on effect size, for any of the PERG parameters; outcome measure was the mean difference between treatments measured at the end of treatment period; 2 × 2 cross-over design two-sided t-test (continuous outcome measure with normal distribution), power > 80%, alpha error 5%, expected mean difference at least 1.4, the actual mean difference 2.1, standard deviation of the period differences for each subject within each sequence is 0.7 (an effect size = 1); we calculated a total sample size of 40 subjects.

Descriptive statistics have been obtained for all variables assessed in the study population. Mean and standard deviation have been used for normally distributed variables, mean and interquartile range for skewed distributions, proportions for categorical variables. Logarithmic transformation has been applied to skewed variables, whenever relevant to achieve normality.

Multilevel generalized linear (MGL) models were applied to take into account the repeated measures within-patient structure of the data. In fact, each eye of each patient was measured three times: at baseline, at the end of period 1, at the end of period 2; therefore, for each patient, 6 correlated observations were available); MGL models allow to take into account the within-patient correlation of values, and calculate the appropriate variance. More specifically, in our models the dependent variables were PERG values, the effect size was the mean difference (measured at the end of treatment period) between the two treatments, the fixed effect sequence and treatment (and their interaction), and the random effects were patient and eye. The carry-over was tested by means of an interaction term between sequence and treatment; if significant, only data from first period were used. Predictions derived from the statistical models were used to estimate effect size and its 95% confidence intervals. In all cases, two-tailed tests were applied. A p-value < 0.05 were considered significant.

## Results

Forty-two patients were enrolled and signed informed consent, but two patients left the clinical trial in the second follow-up period (T4–T8): the first, a woman, due to the onset of an orthopedic problem was unable to be present to the 8 month visit; the second, a man, withdrew consent after the second visit because he did not want to repeat all the required examinations (Fig. 1).

A total of 40 Caucasian subjects completed the study: 21 in group A and 19 in group B. The overall median age was 66.7 [61.5–73.5] years, 21 patients (52.5%) were male; most patients were POAG (35, 87.5%); twenty-two (55%) patients had one or more systemic diseases, in particular eleven (27.5%) subjects had systemic hypertension, seven (17.5%) hypercholesterolaemia, six (15%) vascular disorders (i.e. acute myocardial or cerebral infarction, cardiomyopathy), five (12.5%) diabetes (Table 1). At baseline, most patients (85%) had an early glaucomatous visual field defect with a pathological cup/disc ratio, visual acuity was very good and intra-ocular pressure was generally well controlled (Table 2).

**Table 1:** Baseline demographic data of group A (current topical therapy + PEA 600 mg one tablet a day, in the first period) and group B (current topical therapy alone, in the first period).

	Overall group (40 patients)	Group A (21 patients)	Group B (19 patients)
**Age (median [IQR] years)**	66.7 [61.5–73.5]	67.6 [62.3–75.5]	66.7 [55.7–70]
**Gender (number, %)**			
Male	21 (52.5)	10 (47.6)	11 (57.9)
Female	19 (47.5)	11 (52.4)	8 (42.1)
**Diagnosis**			
POAG (N, %)	35 (87.5)	18 (85.7)	17 (89.5)
NTG (N, %)	5 (12.5)	3 (14.3)	2 (10.5)
**Concomitant systemic diseases (N, %)**			
Systemic hypertension	11 (27.5)	7 (33.3)	4 (21.1)
Hypercholesterolaemia	7 (17.5)	2 (9.5)	5 (26.3)
Vascular disorders	6 (15)	3 (14.3)	3 (15.8)
Diabetes	5 (12.5)	3 (14.3)	2 (10.5)

**Table 2:** Baseline clinical characteristics of group A (current topical therapy + PEA 600 mg one tablet a day, in the first period) and group B (current topical therapy alone, in the first period) (RE = right eye, LE = left eye).

	Overall group (40 patients)		Group A (21 patients)		Group B (19 patients)	
	RE	LE	RE	LE	RE	LE
Visual acuity (decimals)	10 [10–10]	10 [9–10]	10 [10–10]	10 [9–10]	10 [10–10]	10 [10–10]
IOP (mmHg)	14.5 [12.5–16]	14.5 [12–16]	14[12–16]	15[12–16]	15 [13–16]	14 [12–16]
Vertical c/d ratio	0.7 [0.6–0.8]	0.7 [0.7–0.8]	0.7 [0.6–0.8]	0.7 [0.7–0.8]	0.7 [0.6–0.8]	0.7 [0.7–0.8]
**Visual field**						
MD (decibels)	− 2.1 [− 4.2 to − 1.2]	− 3.8[− 6.2 to − 1.5]	− 2.2 [− 3.8 to − 1.5]	− 4.1 [− 5.8 to − 0.9]	− 1.9 [− 4.7 to − 1.1]	− 3.7 [− 6.7 to − 1.9]
PSD (dB)	2.7 [2–5.9]	2.7 [1.9–8.7]	2.8 [2.2–4.5]	2.4 [1.9–8.9]	2.6 [1.6–7.1]	3.3 [1.9–6.6]
**Pattern electroretinogram (P-ERG)**						
Amplitude Amp-p50 (μV)	1.7 [0.8–2.1]	1.1 [0.7–1.9]	1.7 [0.8–2.1]	1.1 [0.7–2.1]	1.6 [1–2.1]	1.2 [0.6–1.9]
Latency Lat-p50 (msec)	56.5 [53–61]	57 [54–59.5]	57 [54–61]	59 [56–60]	55 [52–61]	55 [52–59]
Amplitude Amp-n95 (μV)	2.7 [1.6–3.6]	2.7 [1.4–3.8]	2.6 [1.4–3.5]	2.6 [1.5–3.4]	2.7 [1.9–3.8]	3.5 [1.3–5]
Latency Lat-n95 (msec)	102.5 [98–107.5]	100 [96.5–107.5]	102 [96–107]	100 [96–107]	103 [98–108]	100 [97–109]
**Quality of life (NEI-VFQ25)**						
General health (GH) (score)	50 [50–75]		50		50 [50–75]	
General vision (GV) (score)	60 [60–60]		60 [40–60]		60 [60–60]	
Total mean (score)	65 [60–75]		63.1 [57.5–73.8]		65 [60–75]	

All values are expressed as median [IQR].

Baseline demographic and clinical characteristics were well balanced between the two arms (Tables 1 and 2).

### Function

#### Electrophysiology

**Figure 3:**
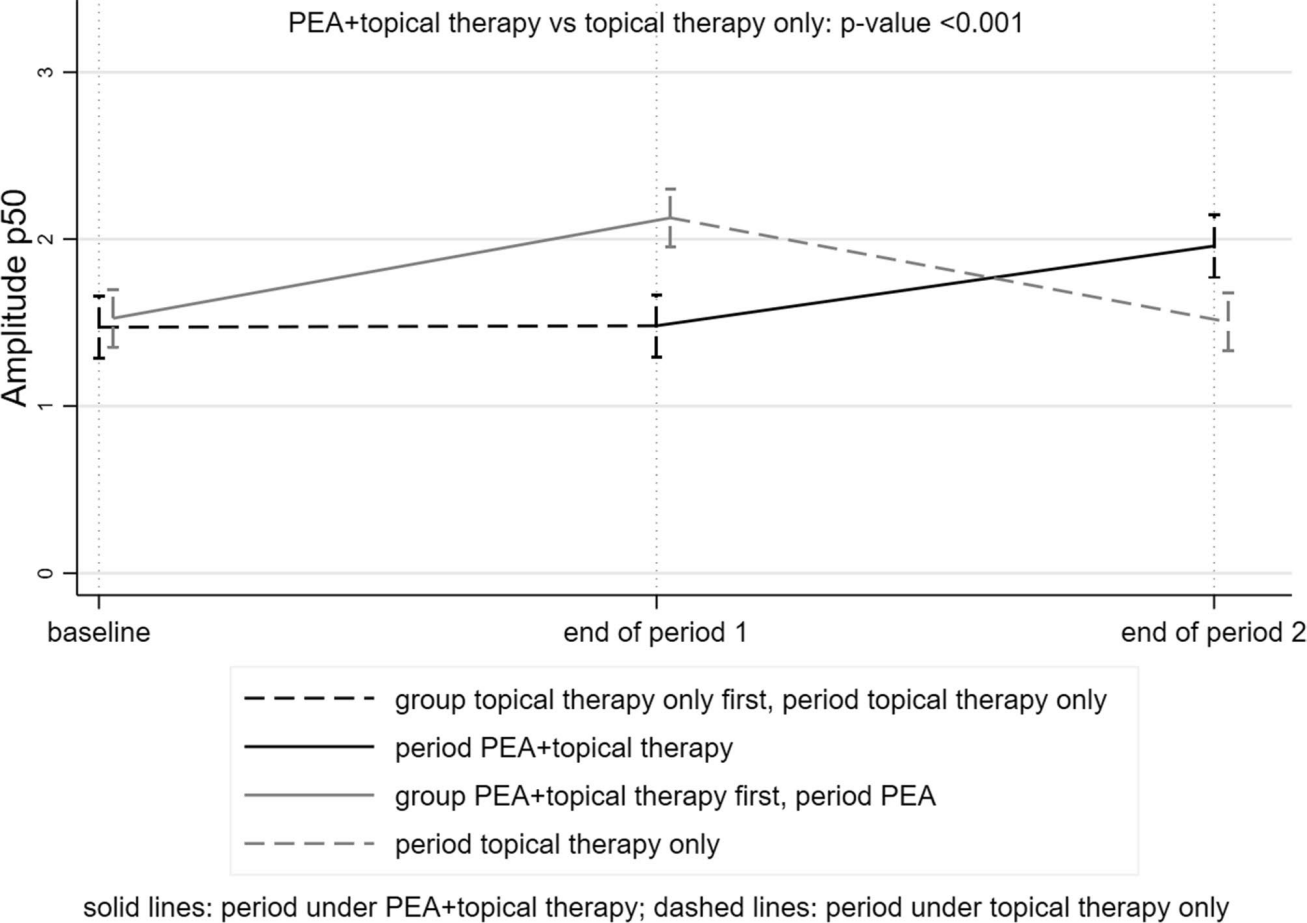
Trend of the Amplitude P50 wave in the topical therapy + tablet intake period (solid lines) and in the topical therapy alone period (dashed lines). At the end of the period of the intake, patients also taking PEA 600 mg had higher P50-wave amplitude.

**Figure 4:**
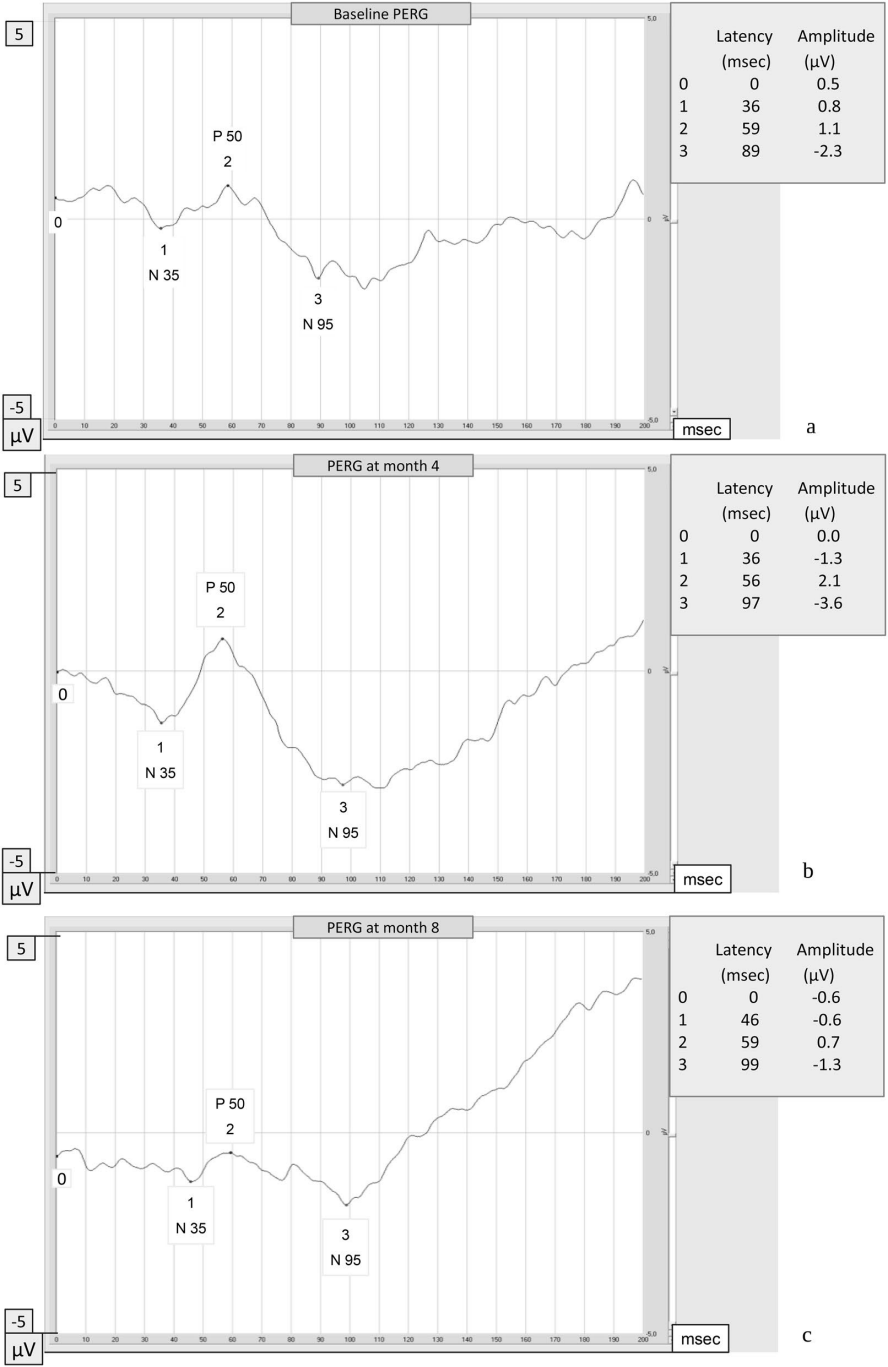
Change of PERG in a patient randomized to group A: (a) baseline PERG (current topical therapy alone); (b) PERG at month 4 (at the end of the period of current topical therapy + PEA 600 mg one tablet a day's assumption); and (c) PERG at month 8 (at the end of the period in which the patient returned to topical therapy alone). (This example is an extreme case where the changes in amplitude are very evident and impressive).

Regarding the transient PERG examination, at the end of the intake period, patients who also took PEA 600 mg had P50-wave amplitude of 0.56 μV (95% CI 0.30–0.73) higher than those on topical therapy alone (p < 0.001) (Tables 3 and 4, Fig. 3). Figure 4 shows the changes in PERG over time in a patient randomized to group A.

**Table 3:** IOP, visual field (VF) and pattern electroretinogram (PERG) examinations over the time by group (group A: current topical therapy + PEA 600 mg one tablet a day, in the first period, and group B: current topical therapy alone, in the first period) (RE = right eye, LE = left eye).

Group	T0 (baseline)	T4 (month 4)	T8 (month 8)	p-value
**IOP (mmHg)**				
A				< 0.001
RE	14 [12–16]	***13 [11–14] ***	14 [12–17]	
LE	15 [12–16]	***13 [12–15]***	14 [13–16]	
B				
RE	15 [13–16]	15 [14–16]	***14 [11–15] ***	
LE	14 [12–16]	15 [13–16]	***13 [12–15]***	
**Visual field**				
MD (dB)				
A				0.642
RE	− 2.2 [− 3.8 to − 1.5]	***− 2.5 [−4.4 to − 1.2]***	− 1.9 [− 3 to − 1.2]	
LE	− 4.1 [−5.7 to − 0.9]	***− 3.3 [− 4.7 to − 1.5]***	− 1.5 [−5.2 to − 0.3]	
B				
RE	− 1.9 [− 4.7 to − 1.1]	− 1.8 [− 4.2 to − 1.2]	***− 2 [− 2.5 to − 1.4]***	
LE	− 3.7 [− 6.7 to − 1.9]	− 3.7 [− 7.1 to − 2.2]	***− 3.6 [− 5.7 to − 2.5]***	
PSD (dB)				
A				0.312
RE	2.8 [2.2–4.5]	***2.5 [1.9–4.9]***	2.5 [2–3.1]	
LE	2.4 [1.9–8.9]	***2.2 [1.7–4]***	1.9 [1.7–2.3]	
B				
RE	2.7 [1.6–7.1]	2.1 [1.7–4.2]	***1.9 [1.7–2.3]***	
LE	3.3 [1.9–6.6]	3.8 [2.4–5.7]	***3.2 [1.9–5.9]***	
**Pattern electroretinogram (P-ERG)**				
P50-wave amplitude (μV)				
A				< 0.001
RE	1.7 [0.8–2.1]	***1.9 [1.3–2.6]***	1.7 [0.8–2.1]	
LE	1.1 [0.7–2.1]	***2.1 [1.4–2.8]***	1 [0.7–2.1]	
B				
RE	1.6 [1–2.1]	1.5 [1.2–1.9]	***1.8 [1.4–2.3]***	
LE	1.2 [0.6–1.9]	1.3 [1.1–1.8]	***1.6 [1.4–2.5]***	
P50-wave Latency (ms)				
A				0.469
RE	57 [54–61]	***55 [54–57]***	59.5 [55–61]	
LE	59 [56–60]	***55 [53–59]***	59 [56–62]	
B				
RE	55 [52–61]	56 [53–61]	***57 [53–61]***	
LE	55 [52–59]	57 [54–62]	***58.5 [53–62]***	
N95-wave Amplitude (μV)				
A				0.257
RE	2.6 [1.4–3.5]	***3.1 [1.8–3.5]***	2.5 [1.7–4.2]	
LE	2.6 [1.5–3.4]	***3.5 [2–3.8]***	2.3 [1.7–3.3]	
B				
RE	2.7 [1.9–3.8]	3 [2.1–3.6]	***2.9 [1.6–4.3]***	
LE	3.5 [1.3–5]	2 [1.6–2.9]	***2.6 [2.1–3.2]***	
N95-wave latency (ms)				
A				0.173
RE	102 [96–107]	***101 [93–108]***	102.5 [95–106.5]	
LE	100 [96–107]	***98 [95–100]***	104 [100–109]	
B				
RE	103 [98–108]	100 [95–105]	***102.5 [95–105]***	
LE	100 [97–109]	98 [95–108]	***102.5 [100–108]***	
**Quality of life (NEI-VFQ 25)**				
General health (GH) (score)				
A	50	***50 [25–75]***	50 [25–50]	0.05
B	50 [50–75]	50	***50 [50–75]***	
General vision (GV) (score)				
A	60 [40–70]	***60 [40–60]***	60 [60–60]	0.19
B	60 [60–60]	60 [40–60]	***60 [60–60]***	
Total mean (score)				
A	63.1 [57.5–73.7]	***62.5 [61.2–76.3]***	60 [48.7–71.3]	< 0.001
B	65 [60–75]	63.7 [57.5–70]	***67.5 [62.5–81.2]***	

All values are expressed as median [IQR]. The values obtained at the end of the PEA 600 mg intake period are reported in bold and italics. P-values refer to the comparison between treatments (see text for statistical analyses methods).

**Table 4:** Difference between periods when patients took PEA + current topical therapy, and periods with current topical therapy alone.

Parameter	Mean difference [95% CI]	p-value
Intraocular pressure (mmHg)	− 1.58 [− 2.00 to 1.16]	** < 0.001**
NEI-VFQ total mean (score)	+ 6.89 [3.98 to 9.80]	** < 0.001**
GH (score)	+ 3.73 [− 0.11 to 7.58]	0.057
GV (score)	+ 1.49 [− 0.73 to 3.70]	0.19
Visual field MD (dB)	− 0.09 [− 0.47 to 0.29]	0.642
Visual field PSD (dB)	+ 0.13 [− 0.12 to 0.38]	0.312
P50-wave amplitude (μV)	+ 0.56 [0.30 to 0.73]	** < 0.001**
P50-wave latency (ms	− 0.64 [− 2.39 to 1.10]	0.469
N95-wave amplitude (μV)	+ 0.24 [− 0.17 to 0.65]	0.257
N95-wave latency (ms)	− 1.60 [− 3.91 to 0.70]	0.173

A slightly higher N95-wave amplitude and a slightly shorter peak time for both P50 and N95 were recorded when patients took PEA without reaching statistical significance.

#### Visual field

Mean deviation and Pattern standard deviation were also not statistically different between the periods in which patients took PEA or not (Tables 3 and 4).

### IOP

**Figure 5:**
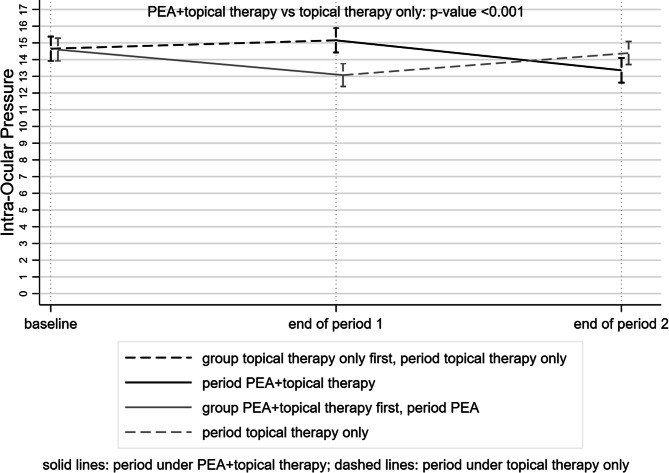
Trend of the intraocular pressure (IOP) in the topical therapy + tablet intake period (solid lines) and in the topical therapy alone period (dashed lines). At the end of the period of the intake, patients also taking PEA 600 mg had lower IOP.

The mean IOP was 1.58 mmHg (95% CI − 2.00 to 1.16) lower when patients were on PEA therapy (p < 0.001) (Tables 3 and 4, Fig. 5).

### Quality of life

During the period in which PEA 600 mg was taken, patients had on average 6.89 (95% CI 3.98–9.8) higher NEI-VFQ25 scores (p < 0.001) (Tables 3 and 4).

## Discussion

The primary objective of the present study was to evaluate the effects of PEA 600 mg a day on PERG examination after four months of daily therapy. The findings of our study suggest a potential neuromodulatory effect of PEA documented by an increase in the amplitudes of the P50 wave.

Glaucoma is a neurodegenerative process involving both the eye and the central nervous system along with reactive glia, synaptic connectivity and axonal transport, neurotrophic factor deprivation, pro-apoptotic signaling activation of neurotransmitters and neuromodulators, as well as excitotoxicity and oxidative stress^6,7,12,31–33^.

PEA has been demonstrated to protect ganglion cells by modulating the glutamate-excitotoxicity^19,20,34^, and acting as a neuromodulator through anti-inflammatory and antioxidant actions^35^. Therefore PEA acts as a neuroprotective and neuromodulating agent able to reduce the ganglion cells neurotoxicity due to different pathogenetic mechanisms involving not only the retina but the entire visual pathway.

The effects of PEA attenuate pro-inflammatory mediators and/or increase anti-inflammatory mediators^36^ acting on PPAR-*α* and PPAR-*γ* (mouse model), thus restoring any neuroinflammation induced downregulation of these receptors^37^.

Oxidative stress and ischemia could affect the aging endothelium showing significant changes in its function. One of the most important factors that is able to regulate aging processes, cellular metabolism^38^ and in neuronal activity^39^ is the Adenosine monophosphate–activated protein kinase (AMPK). This molecule through the modulation of the signaling type 2 cannabinoid receptor reduces oxidative stress in brain injury^40^, inhibiting ROS production induced by mitochondrial dysfunction, and NADPH oxidase. AMPK also inhibits the production of pro-inflammatory factors, preventing endothelial dysfunction by increasing the bioavailability of nitric oxide^41^. Interestingly, AMPK is expressed in TM and is one of the regulators of its motility and homeostasis^42^.

The molecules currently registered for clinical use as neuroprotective agents are CoQ10 and citicoline. CoQ10 (a component of the mitochondrial respiratory chain that plays a role in mitochondrial oxidative phosphorylation) associated with vitamin E topical administration in open-angle glaucoma patients has positive effects on inner retinal function, shown by improved pattern electroretinogram (PERG)^43^. Citicoline is involved in phospholipidis membrane synthesis; it acts by inhibiting dopamine reuptake, increasing levels of dopamine, noradrenaline, serotonin and acetylcholine ^44,45^. Citicoline has protective effects in cerebrovascular and neurodegenerative diseases ^8^: it has been demonstrated to improve PERG response and visual field examination in glaucoma patients treated with intramuscular citicoline in a 8-year follow up study^45^.

Our study differs from previous experiences in that we examined the potential effects of a molecule on patients with stable glaucoma and not on patients with clinical worsening, since we aimed to identify interventions that could potentially prevent neurological damage rather than cure it after it has occurred.

We have chosen an objective outcome measurement (electrophysiological examination) and not the visual field, since the latter could be influenced by patients' well-being.

Our study noted some marginal effects on the visual field. This was not a problem since the possible effect on the visual field was a secondary, exploratory aim, and the study was not powered to detect this. Moreover, the beneficial effects on VF are expected for a longer follow-up period.

It is important to underline that ours is the first study on neuroprotection/neuromodulation in glaucoma which considered the impact of a potentially neuroprotective agent on quality of life^8^. The impact of vision-related quality of life on glaucoma patients is widely recognized: our study suggests that vision-related quality of life may change, improving, while taking one tablet a day of PEA, even if the absence of a placebo group could be a limit in the quality of life assessment. However, a specific study aimed and powered at assessing the quality of life should be done to confirm our data.

Finally, we noticed a significant effect on IOP reduction when taking PEA. Some clinical trials have investigated the effects of PEA on IOP: Costagliola et al.^46^ treated normal tension glaucoma patients with PEA 600 mg a day for 6 months, recording a significant IOP reduction (from 14.4 ± 3.2 mm Hg to 11.1 ± 4.3 mmHg, p < 0.01) at the end of the follow up; similar data have been reported for a shorter period of therapy (two months) by Gagliano^15^ in open angle glaucoma and ocular hypertensive patients. We observed a reduction in IOP of more than 1 mmHg, which may seem like a small reduction: however, the Early Manifest Glaucoma Trial (EMGT) pointed out a 10% reduction in the risk of progression for every mmHg of IOP reduction^2^, so it is still clinically relevant since IOP-mediated ganglion cell dysfunction may be reversible^47^.

Endocannabinoids can provide a direct as well as indirect effect on IOP regulation; in a mouse model it increases the outflow though conventional pathways^48^. Using a porcine anterior segment-perfused organ culture mode, PEA caused a concentration-dependent increase of outflow facility, with the maximum effect achieved at a low concentration of 30 nM^16^. In human eyes, CB1 receptors were found in the Schlemm canal and in the trabecular meshwork, while CB2 receptors were found in the same structure of a porcine model, both receptors have the property of modulating trabecular meshwork cells behaviour by promoting severals modifications^49^. PEA has no effect on CB1 or CB2 receptors, the effect of PEA could be mediated by an entourage effect, which leads to an increase of the cannabinoid tone^50^. Furthermore, in glaucomatous eyes, due to the lower levels of PEA in choroid and ciliary body, it has been hypothesized that PEA has an active role in the IOP regulation and that PEA levels raise in blood flow and tissues as a response to different insults^51^.

In glaucoma, the conventional outflow path represents the greatest passage of aqueous humor towards the collecting channels. The functioning of its cells represents the fundamental pivot of the pathophysiology of the outflow^52^. During glaucoma there is a progressive morphological—functional decay of this outflow pathway and in particular of its endothelial cells: recently Saccà et al. turned their attention to this topic, stating that the proapototic signals that initiate the apoptotic process of the retinal ganglion cells may start from this tissue^53^.

In this context, PEA has an improving effect on endothelial dysfunction^54^ thus protecting cells and improving their homeostasis perhaps also through the involvement of epoxyeicosatrienoic acids and renin angiotensin system^55^. In addition, PEA treatment reduces blood vessel damage, proinflammatory cytokines production and Interleukin 1 beta, the inducible nitric oxide synthase and polymerase formation, nuclear factor kappa-B expression and apoptosis activation attenuating the inflammatory process^55^.

Some observations on the results obtained from our research should be emphasized.

First of all, the visual field examination is the most important test for correct management and follow-up of glaucoma; clinicians often have to deal with discrepancy of structural and functional results. It should be remembered that VF is a patient-dependent exam, which could sometimes produce results that doesn’t correspond to reality, especially when the patient has a very severe defect, poor retinal function or cognitive impairment that causes lack of attention and ability to perform different tasks.

Secondly, the use of electrophysiology in glaucoma is increasingly considered useful in the correct management of this pathology, since it is more objective than VF. However, it requires a skilled examiner.

Third, to date the ideal neuroprotector has not been established: further data on the mechanism of action and neuroprotective potential will require larger, long-term studies.

Regarding pros and cons of our study: the biggest limitation is the limited sample size, and a short follow-up, that do not allow us to infer efficacy on a broader patient population; another limitation is that only PERG was examined, so it is not possible to say whether the change in PERG was in ganglion cells, or it was a change in cellular function distal to the ganglion cells. The main strength of the study is the design: randomized, cross over and single blind.

In conclusion, the exact pathogenesis of glaucoma is not fully understood: in vitro studies have shown that the ocular hypertension affects the RGC population, but it spares non-RGC neurons located in the ganglion cell layer of the retina^56^. Aside from IOP reduction, endogenous and exogenous neuroprotective factors are involved in RGC survival, therefore a single molecule may not be enough to protect RGC since different mechanism of action are involved in RGC death.

PEA has shown promising effects on both PERG and quality of life; it should be emphasized that the IOP reduction itself acts as indirect neuroprotection, and PEA is able to significantly reduce the IOP.
